# *Achyranthes bidentata* Polypeptides Reduces Oxidative Stress and Exerts Protective Effects against Myocardial Ischemic/Reperfusion Injury in Rats

**DOI:** 10.3390/ijms141019792

**Published:** 2013-09-30

**Authors:** Ru Tie, Lele Ji, Ying Nan, Wenqing Wang, Xiangyan Liang, Fei Tian, Wenjuan Xing, Miaozhang Zhu, Rong Li, Haifeng Zhang

**Affiliations:** 1 Experiment Teaching Center, Fourth Military Medical University, Xi’an 710032, China; E-Mails: sophia_ru@163.com (R.T.); seasonglad@126.com (L.J.); liangxiangyan@126.com (X.L.); shilin8@fmmu.edu.cn (F.T.); 2 Department of Physiology, Xi’an Medical University, Xi’an 710021, China; E-Mail: nanying2002419@163.com; 3 Department of Hematology, Tangdu Hospital, Fourth Military Medical University, Xi’an 710038, China; E-Mail: rongli.li09@gmail.com; 4 Department of Physiology, Fourth Military Medical University, Xi’an 710032, China; E-Mail: xwjfmmu@126.com; 5 Department of Geratology, Xijing Hospital, Fourth Military Medical University, Xi’an 710032, China

**Keywords:** *Achyranthes bidentata* polypeptides, oxidative stress, myocardial ischemia/reperfusion, apoptosis

## Abstract

*Achyranthes bidentata*, a Chinese medicinal herb, is reported to be neuroprotective. However, its role in cardioprotection remains largely unknown. Our present study aimed to investigate the effects of *Achyranthes bidentata* polypeptides (ABPP) preconditioning on myocardial ischemia/reperfusion (MI/R) injury and to test the possible mechanisms. Rats were treated with ABPP (10 mg/kg/d, i.p.) or saline once daily for one week. Afterward, all the animals were subjected to 30 min of myocardial ischemia followed by 4 h of reperfusion. ABPP preconditioning for one week significantly improved cardiac function following MI/R. Meanwhile, ABPP reduced infarct size, plasma creatine kinase (CK)/lactate dehydrogenase (LDH) activities and myocardial apoptosis at the end of reperfusion in rat hearts. Moreover, ABPP preconditioning significantly inhibited superoxide generation, gp91^phox^ expression, malonaldialdehyde formation and enhanced superoxide dismutase activity in I/R hearts. Furthermore, ABPP treatment inhibited PTEN expression and increased Akt phosphorylation in I/R rat heart. PI3K inhibitor wortmannin blocked Akt activation, and abolished ABPP-stimulated anti-oxidant effect and cardioprotection. Our study demonstrated for the first time that ABPP reduces oxidative stress and exerts cardioprotection against MI/R injury in rats. Inhibition of PTEN and activation of Akt may contribute to the anti-oxidant capacity and cardioprotection of ABPP.

## 1. Introduction

Myocardial infarction (MI) remains one of the leading causes of death and disability throughout the world. MI and related complications (e.g., heart failure) are great socio-economic burdens to society and healthcare systems. Blood flow restoration through the culprit coronary artery is necessary to save endangered myocardium after acute myocardial infarction. It is well established that early reperfusion limits infarct size and improves clinical outcome of patients with acute MI. The process of restoring blood flow to the ischemic myocardium, however, can induce injury, myocardial reperfusion injury, which can paradoxically reduce the beneficial effects of myocardial reperfusion and cause contractile dysfunction and cellular damage [1–3]. Despite intensive research, therapeutic strategies are limited and there is, as yet, no effective routine, generally accepted and promising approaches for myocardial ischemia/reperfusion (MI/R) injury.

In recent years, there is a growing interest in the treatment of MI/R-induced cardiac dysfunction with plant-based therapy including traditional Chinese medicine, for which extensive experience has been accumulated over thousands of years. *Achyranthes bidentata* Blume (*A. bidentata*), a commonly prescribed traditional Chinese herb, occupies an important position in traditional Chinese stroke therapy owing to the property of promoting the circulation of blood and removing stasis [4]. However, the influence of *Achyranthes bidentata* polypeptides (ABPP) on MI/R injury in rat has been largely unexplored until now.

Strong evidence exists that increased oxidative stress, which oxidizes biological macromolecules and impairs cell functions, is a major pathogenic factor in MI/R injury [5]. Oxidative stress is usually associated with increased formation of reactive oxygen species (ROS). Oxygen radicals could react with membrane phospholipids, proteins, nucleic acids and other cellular components, acting on the membrane fatty acids, further generate lipid free radicals and lipid peroxides, and impair cell structure and function, leading to cell damage. Evidence has also shown that MI/R promotes excess generation of highly ROS and causes oxidative stress which further exacerbates the development and progression of cardiopathy and its complications [6]. Thus, oxidative stress acts as a major mechanism in myocardial vulnerability to MI/R. However, the effects of ABPP on oxidative stress following MI/R remain unknown.

It is widely accepted that activation of the phosphatidylinositol-3-kinase (PI3K)/protein kinase B (Akt) pathway regulates myocardial oxidative stress and contributes to the cardioprotection against MI/R injury [7]. Phosphatase and tensin homolog deleted on chromosome 10 (PTEN), originally identified as a negative regulator of PI3K signaling, also plays an important role in regulating oxidative stress [8,9]. Previous study has indicated that ABPP-E4, an active fraction from ABPP, prevented apoptosis induced by serum deprivation in SH-SY5Y cells through PI3K/Akt signaling [10]. Nevertheless, whether ABPP regulates PTEN and Akt signaling following MI/R *in vivo* remains unknown.

Therefore, the present study was designed to investigate whether ABPP exerts cardioprotection against MI/R injury and the underlying mechanisms.

## 2. Results and Discussion

### 2.1. *Achyranthes bidentata* Polypeptides Alleviated MI/R-Induced Cardiac Dysfunction

**Figure 1:**
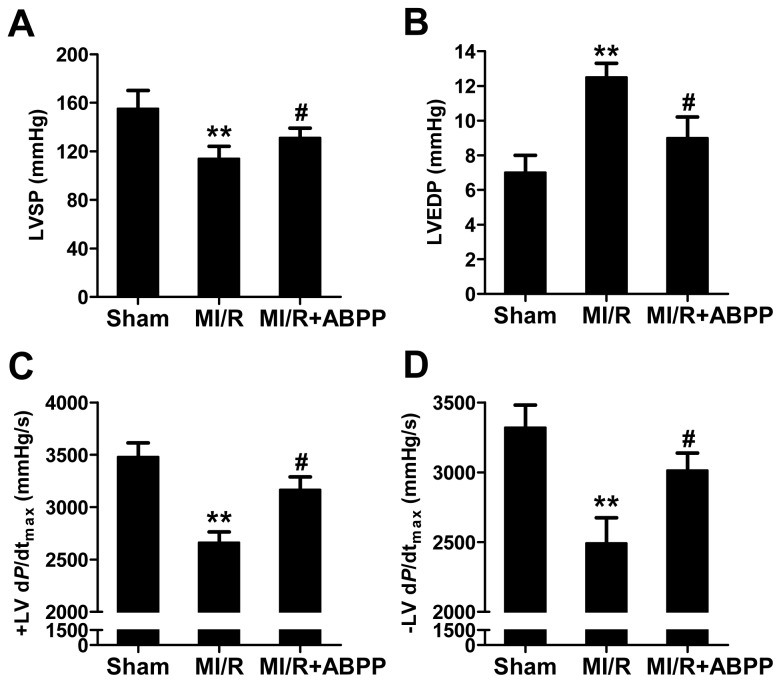
*Achyranthes bidentata* polypeptides (ABPP) preconditioning increased cardiac functions in rats subjected to myocardial ischemia/reperfusion. (**A**) LVSP, left ventricular systolic pressure; (**B**) LVEDP, left ventricular end diastolic pressure; (**C**) +LV d*P*/d*t*_max_, the instantaneous first derivation of left ventricle pressure; (**D**) −LVdP/d*t*_max_. MI/R, myocardial ischemia/reperfusion (30 min/4 h); Sham, sham-operated; ABPP, *Achyranthes bidentata* polypeptides. Values presented are means ± SEM. *n* = 8/group. **p < 0.01 *versus* Sham, ^#^*p* < 0.05 *versus* MI/R.

As shown in Figure 1, MI/R significantly impaired cardiac functions as evidenced by decreased left ventricular systolic pressure (LVSP) and the instantaneous first derivation of left ventricle pressure (± LV d*P*/d*t*_max_) and increased LVEDP after 4 h of reperfusion (*n* = 8, *p* < 0.01). ABPP preconditioning improved cardiac function in MI/R rats, indicating that ABPP pretreatment markedly increased cardiac function during MI/R.

### 2.2. *Achyranthes bidentata* Polypeptides Alleviated I/R-Induced Myocardial Injury

**Figure 2:**
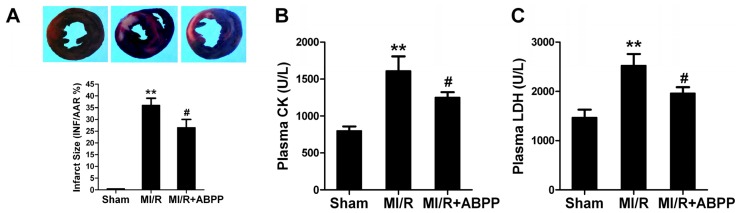
*Achyranthes bidentata* polypeptides (ABPP) preconditioning reduced infarct size, plasma creatine kinase (CK) and lactate dehydrogenase activities (LDH) in rats subjected to myocardial ischemia/reperfusion. (**A**) Myocardial infarct size expressed as percentage of area-at-risk (AAR); (**B**) Plasma creatine kinase (CK) levels; (**C**) Plasma lactate dehydrogenase (LDH) levels. MI/R, myocardial ischemia/reperfusion (30 min/4 h); Sham, sham-operated; ABPP, *Achyranthes bidentata* polypeptides. Values presented are means ± SEM. *n* = 8/group. **p < 0.01 *versus* Sham MI/R, ^#^*p* < 0.05 *versus* MI/R.

Thirty minutes of ischemia and 4 h of reperfusion resulted in myocardial injury, consistent with previously reported results, as evidenced by increased infarct size (Figure 2A). Compared with MI/R group, ABPP preconditioned rats showed a significantly reduction in myocardial infarct size (36.0 ± 3.0 *vs*. 26.5 ± 3.5% in MI/R group, *n* = 8, *p* < 0.05).

We next detected plasma LDH and CK activities to evaluate myocardial necrosis in rat with MI/R. At the end of the experiment, plasma CK and LDH activities in MI/R group were increased to 1616 ± 198 and 2523 ± 232 U/L, respectively (*n* = 8, *p* < 0.01 *vs*. Sham, Figure 2B,C). Interestingly, in ABPP-preconditioned I/R rats, plasma CK and LDH levels were decreased to 1251 ± 72 and 1961 ± 122 U/L (*p* < 0.05 *vs*. MI/R). These results suggested that ABPP preconditioning decreased myocardial necrosis in I/R rats.

**Figure 3:**
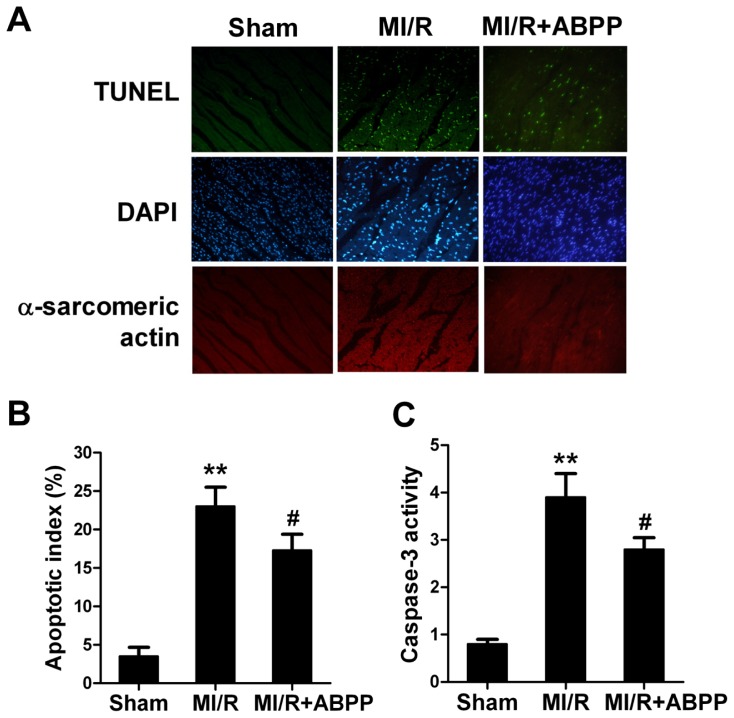
*Achyranthes bidentata* polypeptides (ABPP) preconditioning reduced myocardial apoptotic index and caspase-3 activity in rats subjected to myocardial ischemia/reperfusion. (**A**) Top: representative photomicrographs of *in situ* detection of apoptotic myocytes by terminal deoxynucleotidyl nickend labeling (TUNEL) staining in ischemic heart tissue from rats subjected to 30 min of ischemia and 4 h of reperfusion. Green fluorescence shows TUNEL-positive nuclei; Blue fluorescence shows nuclei of total cardiomyocytes. Bottom: percentage of TUNEL-positive nuclei in heart tissue sections; (**B**) Myocardial caspase-3 activity. MI/R, myocardial ischaemia/reperfusion (30 min/4 h); Sham, sham-operated; ABPP, *Achyranthes bidentata* polypeptides. Values presented are means ± SEM. *n* = 8/group. **p < 0.01 *versus* Sham, ^#^*p* < 0.05 *versus* MI/R.

Apoptosis is a major form of cell death after a short period of ischemia followed by reperfusion. Here we observed the effects of ABPP on I/R-induced cardiomyocyte apoptosis. 4 h after reperfusion, a significant number of TUNEL-positive cells were observed in cardiac tissue (*n* = 8, *p* < 0.01 *vs*. Sham, Figure 3A). ABPP preconditioning exerted a significant anti-apoptotic effect as evidenced by reduced TUNEL-positive staining in rats (*p* < 0.05). To further examine whether ABPP preconditioning could decrease myocardial apoptosis, myocardial caspase-3 activity, a final common pathway in caspase-dependent apoptosis, was determined. MI/R increased caspase-3 activity in rats compared with Sham group (*n* = 8, *p* < 0.01, Figure 3B). ABPP treatment resulted in a significant reduction in caspase-3 activity in I/R rats (*p* < 0.05). All these results provided direct evidence that ABPP alleviated I/R-induced myocardial injury in rats.

### 2.3. *Achyranthes bidentata* Polypeptides Decreased Oxidative Stress in I/R Hearts

**Figure 4:**
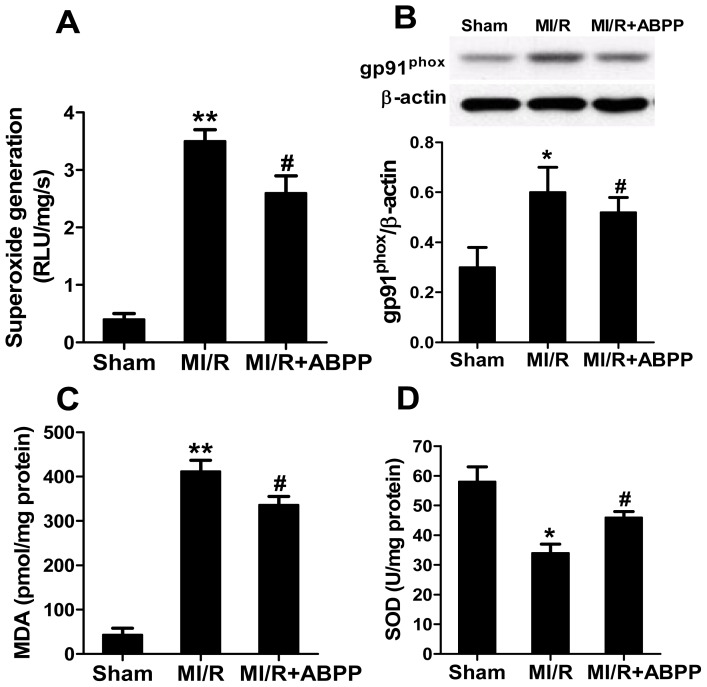
*Achyranthes bidentata* polypeptides (ABPP) preconditioning reduced oxidative stress in rats subjected to myocardial ischemia/reperfusion. (**A**) Cardiac superoxide generation; (**B**) gp91^phox^ expression. Top images: representative blots; (**C**) Myocardial malondialdehyde (MDA) contents; (**D**) Myocardial superoxide dismutase (SOD) activity. MI/R, myocardial ischemia/reperfusion (30 min/4 h); Sham, sham-operated; ABPP, *Achyranthes bidentata* polypeptides. Values presented are means ± SEM. *n* = 8/group. *p < 0.05, **p < 0.01 *versus* Sham, ^#^*p* < 0.05versus MI/R.

As seen in Figure 4A, compared with sham-operated hearts, I/R myocardium showed a significant increase in superoxide content (*p* < 0.01, Figure 4B), and ABPP preconditioning decreased superoxide accumulation (*p* < 0.05 *vs*. MI/R). Afterwards, we determined gp91^phox^ expression, a major component of NADPH oxidase that is the most important superoxide-producing enzyme in I/R heart. As expected, ABPP treatment markedly decreased MI/R-stimulated gp91^phox^ expression (*p* < 0.05, Figure 4B). In addition, MDA was observed as a biomarker to measure the level of oxidative stress. There was a robust increase in MDA production in I/R hearts compared with the sham group, and treatment with ABPP reduced this harmful effect (Figure 4C). In contrast, antioxidant enzyme SOD activity in cardiac tissue was also increased in I/R rats with ABPP preconditioning (Figure 4D). These results demonstrated that ABPP reduced superoxide overproduction and decreased oxidative stress in I/R hearts.

### 2.4. *Achyranthes bidentata* Polypeptides Inhibited PTEN and Activated Akt in I/R Hearts

**Figure 5:**
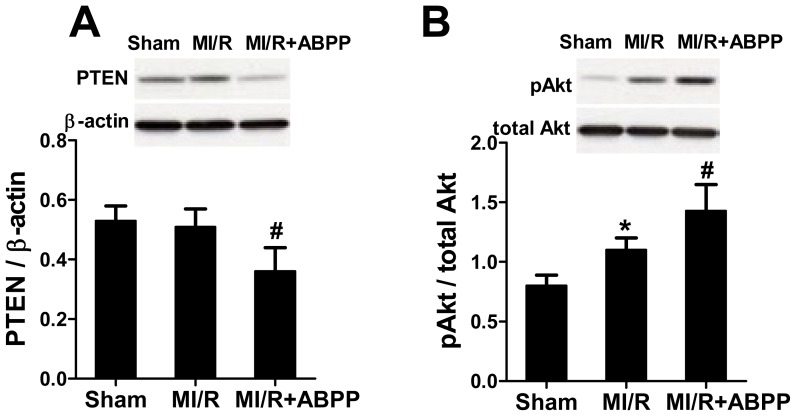
*Achyranthes bidentata* polypeptides (ABPP) preconditioning inhibited PTEN expression and increased Akt phosphorylation in rats subjected to myocardial ischemia/reperfusion. (**A**) PTEN expression; (**B**) Akt expression and phosphorylation. Top images: representative blots; MI/R, myocardial ischemia/reperfusion (30 min/4 h); Sham, sham-operated; ABPP, *Achyranthes bidentata* polypeptides. Values presented are means ± SEM. *n* = 5/group. *p < 0.05 *versus* Sham, ^#^*p* < 0.05 *versus* MI/R.

Akt activation protects against oxidative stress and myocardial I/R injury, whereas increased expression of PTEN has the opposite effect, at least in part, through suppressing Akt [5,8,11]. As shown in Figure 5A, there was no significant difference in PTEN expression between Sham and MI/R group. However, in ABPP preconditioned rats PTEN expression in cardiac tissue was markedly decreased (*p* < 0.05 *vs*. MI/R group, Figure 5A), indicating that ABPP treatment inhibited cardiac PTEN. Moreover, our data revealed that I/R increased Akt phosphorylation, and this effect was markedly augmented by ABPP treatment (*p* < 0.05 *vs*. MI/R group, Figure 5B). These results indicated that ABPP preconditioning inhibited PTEN expression and thus activated survival Akt signaling following MI/R.

### 2.5. Wortmannin Abolished ABPP-Induced Anti-Oxidative Effect and Cardioprotection

**Figure 6:**
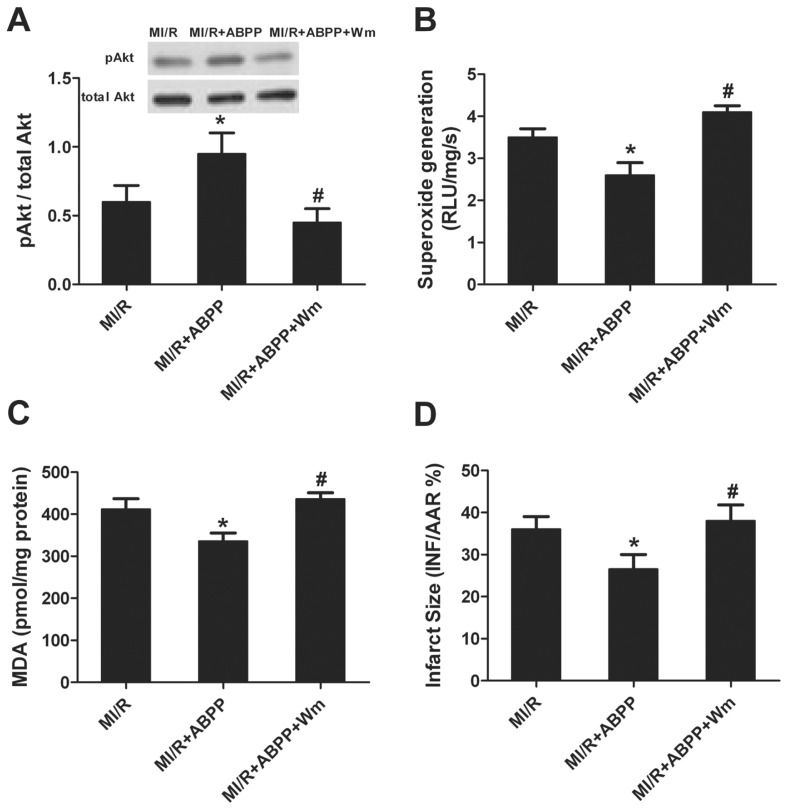
PI3K inhibitor wortmannin inhibited phosphorylation of Akt, increased superoxide generation, malondialdehyde (MDA) and myocardial infarction. (**A**) Phosphorylation of Akt; (**B**) Cardiac superoxide generation; (**C**) Myocardial malondialdehyde (MDA) contents; (**D**) Myocardial infarct size (INF) expressed as percentage of area-at-risk (AAR); MI/R, myocardial ischemia/reperfusion (30 min/4 h); Sham, sham-operated; ABPP, *Achyranthes bidentata* polypeptides; W, wortmannin. Values presented are means ± SEM. *n* = 5–8/group. *p < 0.05 *versus* Sham, ^#^*p* < 0.05 *versus* MI/R.

Our study demonstrated that ABPP preconditioning activated Akt. To determine whether ABPP stimulated cardioprotection through Akt signaling pathway, the PI3K inhibitor, wortmannin, was used. As seen in Figure 6A, there was no significant difference in Akt expression among all groups. Wortmannin markedly inhibited ABPP-enhanced Akt phosphorylation. Moreover, wortmannin significantly blocked the anti-oxidative defense of ABPP as evidence by increased superoxide generation (Figure 6B) and MDA formation (Figure 6C). In addition, myocardial infarct size was increased following wortmannin treatment (Figure 6D), indicating that the cardioprotection of ABPP was also abolished by wortmannin. These data revealed that ABPP alleviates MI/R-induced cardiac injury possibly via the PI3K/Akt signal pathway.

### 2.6. Discussion

Several important observations were made in the present study. First, we have demonstrated for the first time that ABPP preconditioning exerts protective effects against MI/R injury in rats. Second, preconditioning with ABPP acts directly on myocardial oxidative stress reduction in I/R cardiac tissue. Third, inhibition of PTEN and activation of Akt contribute to the anti-oxidant capacity and cardioprotection of ABPP.

*Achyranthes bidentata* Blume is a commonly prescribed Chinese medicinal herb. Previous studies have shown that, *Achyranthes bidentata* polypeptides could improve the recovery of sensory, motor and coordination, and cognitive function in middle cerebral artery occlusion-induced ischemic rats [4]. Moreover, *Achyranthes bidentata* polypeptides has been shown to be effective in prevention of glutamate-induced cell damage in primarily cultured hippocampal neurons and to accelerate peripheral nerve regeneration in a dose-dependent manner [12–14]. Therefore, it is well-accepted that *Achyranthes bidentata* blume is neuroprotective [10,15]. However, the role of *Achyranthes bidentata* in cardioprotection remains unclear. In our present study, pretreatment with ABPP significantly improved cardiac function and alleviated myocardial injury (as evidenced by decreased myocardial infarction, plasma CK/LDH activities and cardiomyocyte apoptosis) in I/R rats, suggesting that ABPP exerts cardioprotection and could be a wide range of effect of cell protection.

ROS have long been recognized to cause oxidative stress and act as the major mediators of MI/R injury. Experimental studies have also demonstrated that ROS released during the early phase of myocardial reperfusion, strongly oxidize cardiomyocytes which have already been damaged by the ischemia [6]. Of the many theories regarding the development of reperfusion injury, the enhanced generation of highly ROS by the heart during the acute reperfusion phase, including superoxide anion, hydrogen peroxide and hydroxyl radical, is an appealing one that is supported by a large foundation of experimental evidence [16–18]. All these suggest that inhibition of oxidative stress may reduce ROS overproduction and exert protective effect against MI/R injury. In the present study, we found that treatment with ABPP directly inhibited superoxide generation. NADPH oxidase is an important source for superoxide anion production. ABPP treatment significantly reduced gp91^phox^ expression, a critical component of NADPH oxidase. The level of oxidative stress in tissue usually correlates with MDA concentration. ABPP treatment also decreased MDA formation in I/R rat heart. Meanwhile, treatment with ABPP significantly improved antioxidant enzyme SOD activity in I/R cardiac tissue. On the basis of these observations, ABPP protects cardiomyocytes against oxidative stress in I/R rat hearts.

Akt is deemed as a key regulator of cell survival. Our previous studies have demonstrated that activation of Akt alleviated cardiac oxidative stress and inhibited myocardial apoptosis, ultimately confer protection against ischemic injury [5,19]. *In vitro* study revealed that *Achyranthes bidentata* inhibits cell apoptosis induced by serum deprivation via PI3K/Akt pathway [10]. This result indicated that ABPP might afford cardioprotection through Akt signaling. In addition, pro-apoptotic protein PTEN, the main negative regulator of PI3K/Akt pathway, has been shown to act as a tumor suppressor whose function includes important roles in regulating oxidative stress [8]. Experimental evidence revealed that cardiomyocyte-specific conditional PTEN deletion limited myocardial infarct size in an *in vivo* model of ischemia/reperfusion injury [20], indicating a detrimental role of PTEN in cardioprotection. However, the effect of *Achyranthes bidentata* on myocardial PTEN has not yet been explored. Data from our present study revealed that ABPP treatment significantly inhibit PTEN expression and increased Akt phosphorylation in I/R cardiac tissue. These results suggested that PTEN and Akt signals were possibly involved in ABPP-induced cardioprotection. To further clarify the role of Akt in ABPP-induced beneficial effects, PI3K inhibitor wortmannin was used. Inhibition of Akt markedly enhanced oxidative stress and increased myocardial infarction, indicating that ABPP exerts anti-oxidative stress and cardioprotection through PI3K/Akt signaling.

Our present study established ABPP as a cardioprotective herb, indicating that it could be a promising effective complementary and alternative medicine in treatment of cardiovascular diseases, especially in preventing diseases and promoting general health and well being. Nonetheless, further studies in the cardioprotection of ABPP are still needed, *i.e*., ABPP used as a treatment strategy after acute myocardial infarction.

## 3. Experimental Section

### 3.1. Preparation and Characterization of ABPP

ABPP was isolated from the aqueous extract of *Achyranthes bidentata* Blume root as previously described [21]. In brief, the plant root was powdered and placed in water at 80 °C. The resulting solution was saturated with ammonium sulfate to 50% saturation, followed by centrifugation at 15,000 rpm for 30 min. The precipitate was discarded, and the supernatant was further saturated with ammonium sulfate to 80% saturation, followed by centrifugation at 15,000 rpm for 30 min. The precipitate formed in the second centrifugation step was dissolved in water to undergo dialysis in 1000 MW cutoff tubing with ultrapure water. All procedures were performed at 48 °C. The dialyzate was then lyophilized to yield the powder of ABPP, which could be dissolved to furnish the desired concentrations prior to use. HPLC analysis provided the quality control for ABPP.

### 3.2. ABPP Treatment

The experiments were performed in adherence with the National Institutes of Health Guidelines for the Use of Laboratory Animals and were approved by the Fourth Military Medical University Committee on Animal Care. Six-week-old male Sprague-Dawley rats (200–220 g) were housed in temperature controlled cages (20–22 °C) with a 12-hour light-dark cycle, and given free access to water and formulated diets. The animals were acclimatized for a 2-week period before starting the protocol. Afterward, the rats were maintained on standard rat diet and treated with ABPP (10 mg/kg/day, i.p., 10 mg/mL) or saline once daily for 1 week. The ABPP solution was prepared with 10 mg ABPP dissolved in 1 mL saline.

### 3.3. Animal Protocols

Rats were fasted overnight and anesthetized through intraperitoneal administration of 60 mg/kg pentobarbital sodium. The animals were subjected to 30 min of myocardial ischemia followed by 4 h of reperfusion. Myocardial ischaemia was produced by exteriorizing the heart with a left thoracic incision followed by making a slipknot (6-0 silk) around left anterior descending (LAD) coronary artery. In sham operated animals the suture was placed beneath the LAD without ligation. Reperfusion was initiated by releasing the ligature and removing the polyethylene-10 tubing. A microcatheter was inserted into LV through right carotid artery to measure the LV pressure. The artery pressure was measured by right femoral artery intubation. Intravenous infusion was executed through left external jugular vein. Hemodynamic data were continuously monitored on a polygraph and simultaneously digitized by using a computer interfaced with an analogue-to-digital converter. Blood samples were drawn from caudal vein before at the end of the reperfusion.

Rats were randomly assigned to three experimental groups: (1) Sham; (2) MI/R; (3) MI/R+ABPP. Sham-operated rats underwent the same surgical procedures with the exception of left anterior descending coronary artery occlusion. Hearts were excised at the end of reperfusion and the tissue from the area-at-risk was harvested. In separate rats, hearts were excised to determine myocardial infarct size. For futher determining the underlying mechnisms, in additional experiments the rats were pretreated by wortmannin (15 μg/kg, *i.v*., 15 min before reperfusion), a PI3K inhibitor.

### 3.4. Determination of Myocardial Infarction and Apoptosis

At the end of 4 h reperfusion, myocardial infarction was determined by means of a double-staining technique and a digital imaging system (infarct area/area-at-risk × 100%). Myocardial apoptosis was analysed by TUNEL (terminal deoxynucleotidyl nickend labeling) assay using an *in situ* cell death detection kit (Roche Molecular Biochemicals, Mannheim, Germany). TUNEL staining for apoptotic cell nuclei and 4′,6-diamino-2-phenylindole staining for all myocardial cell nuclei and α-sarcomeric actin staining for cardiomyocytes as described previously. The index of apoptosis was expressed by number of apoptotic myocytes/the total number of myocytes counted × 100%. The caspase-3 activity of cardiomyocytes was measured by using caspase colorimetric assay kits (Chemicon International, Temecula, CA, USA), as described in our previous study [22].

### 3.5. Determination of Plasma Creatine Kinase (CK) and Lactate Dehydrogenase (LDH)

Blood samples (1 mL) were drawn at 4 h after reperfusion. Plasmacreatine kinase (CK) and lactate dehydrogenase (LDH) activities were measured spectrophotometrically (Beckman DU 640, San Diego, CA, USA) in a blinded manner. All measurements were assayed in duplicate.

### 3.6. Quantification of Superoxide Production

Superoxide production in tissue was measured by lucigenin-enhanced chemiluminescence as described previously [19]. Superoxide production was expressed as relative light units (RLU) per second per milligram heart weight (RLU/mg/s).

### 3.7. Determination of Tissue Malondialdehyde and Superoxide Dismutase

The malondialdehyde (MDA) level and activities of antioxidant enzyme superoxide dismutase (SOD) in heart homogenates were determined spectrophotometrically as previously described [23].

### 3.8. Western Blot Analysis

The expressions of Akt and PTEN and the phosphorylation of Akt were measured using Western blot as described previously [19]. Protein content was determined with BCA protein assay and protein samples were separated by electrophoresis on SDS-PAGE and transferred to a polyvinylidene difluoride membrane. The membranes were blocked with 5% milk and incubated overnight with the appropriate primary antibodies respectively (anti-gp91^phox^, anti-phospho-(p)-Akt, anti-Akt, and anti-PTEN (Cell Signaling Technology, Beverly, MA, USA)), followed by incubation with the corresponding secondary antibodies. The blots were visualized with ECL-plus reagent. Beta-actin was used as the internal loading control.

### 3.9. Statistical Analysis

All values are presented as means ± SEM. Differences were compared by ANOVA followed by Bonferroni correction for *post hoc t* test, where appropriate. Probabilities of <0.05 were considered to be statistically significant. All the statistical tests were performed with the GraphPad Prism software version 5.0 (GraphPad Software, San Diego, CA, USA).

## 4. Conclusions

In conclusion, data from this study demonstrated for the first time that *Achyranthes bidentata* polypeptides preconditioning reduces oxidative stress and alleviates MI/R injury. Inhibition of PTEN and activation of PI3K/Akt signaling, contribute to the anti-oxidant capacity and cardioprotection of ABPP. These findings provide potential benefits of ensuring *Achyranthes bidentata* preconditioning, as a strategy to prevent ischemic heart disease.
